# Sex roles in nest keeping – how information asymmetry contributes to parent‐offspring co‐adaptation

**DOI:** 10.1002/ece3.1976

**Published:** 2016-02-19

**Authors:** Carsten Lucass, Nolwenn Fresneau, Marcel Eens, Wendt Müller

**Affiliations:** ^1^ Department of BiologyBehavioural Ecology and Ecophysiology GroupUniversity of AntwerpUniversiteitsplein 12610AntwerpWilrijkBelgium

**Keywords:** Behavioral reaction norm, co‐evolution, conflict resolution, *Cyanistes caeruleus*, parental care, parent‐offspring conflict

## Abstract

Parental food provisioning and offspring begging influence each other reciprocally. This makes both traits agents and targets of selection, which may ultimately lead to co‐adaptation. The latter may reflect co‐adapted parent and offspring genotypes or could be due to maternal effects. Maternal effects are in turn likely to facilitate in particular mother‐offspring co‐adaptation, further emphasized by the possibility that mothers are sometimes found to be more responsive to offspring need. However, parents may not only differ in their sensitivity, but often play different roles in postnatal care. This potentially impinges on the access to information about offspring need. We here manipulated the information on offspring need as perceived by parents by playing back begging calls at a constant frequency in the nest‐box of blue tits (*Cyanistes caeruleus*). We measured the parental response in provisioning to our treatment, paying particular attention to sex differences in parental roles and whether such differences alter the perception of the intensity of our manipulation. This enabled us to investigate whether an information asymmetry about offspring need exists between parents and how such an asymmetry relates to co‐adaptation between parental provisioning and offspring begging. Our results show that parents indeed differed in the frequency how often they perceived the playback due to the fact that females spent more time with their offspring in the nest box. Correcting for the effective exposure of an adult to the playback, the parental response in provisioning covaried more strongly (positive) with offspring begging intensity, independent of the parental sex, indicating coadaptation on the phenotypic level. Females were not more sensitive to experimentally increased offspring need than males, but they were exposed to more broadcasted begging calls. Therefore, sex differences in access to information about offspring need, due to different parental roles, have the potential to impinge on family conflicts and their resolution.

## Introduction

In a number of species, offspring rely for a given time on pre‐ and postnatal parental care (Clutton‐Brock 1991; Royle et al. 2012). A substantial amount of postnatal parental care consists of food provisioning where parents interact with their offspring that possess private information about their nutritional requirements. Offspring signal these requirements to their parents via begging, to which the latter respond to by providing food. Once the offspring's nutritional requirements are satisfied, they will ultimately reduce their begging intensity and parents will decrease feeding (Kilner and Hinde 2012). In other words, parental provisioning and offspring begging influence each other, and are consequently agents and targets of selection at the same time (Lock et al. 2004). This reciprocal interplay, in combination with the fact that the individual as well as its social environment (i.e. traits of family members) can evolve (Cheverud 2003; Wolf 2003), has led to the hypothesis that parental provisioning and offspring begging should co‐evolve (e.g. Wolf and Brodie 1998). Such intergenerational within‐family co‐adaptation has indeed been found in numerous species (e.g. burrower bugs (*Sehirus cinctus*), Agrawal et al. 2001; mice (*Mus musculus*), Curley et al. 2004; Hager and Johnstone 2003; burying beetles (*Nicrophorus vespilloides*), Lock et al. 2004; rhesus macaques (*Macaca mulatta*), Maestripieri 2004; canaries (*Serinus canaria*), Estramil et al. 2013; Hinde et al. 2009).

Parent‐offspring co‐adaptation may be due to co‐adapted parent and offspring genotypes or reflect (prenatal) maternal effects, which have been hypothesized to adjust offspring begging to parental capacity (Hinde et al. 2009). Since maternal effects are obviously under the control of mothers, this should in particular support mother‐offspring co‐adaptation. However, most previous studies have provided little insight into sex differences in within‐family co‐adaptation in biparental species, as they investigated care traits that are exclusively expressed in females (i.e. uni‐parental maternal care only, Agrawal et al. 2001; contact behaviour, Maestripieri 2004; milk let‐down, Curley et al. 2004). Other studies have used brood weight as a proxy for parental provisioning (Hinde et al. 2009; Estramil et al. 2013), which prevents investigating sex differences in co‐adaptation. However, in great tits (*Parus major*) maternal, but not paternal, provisioning responsiveness toward begging playbacks with a constant interval was positively related to the mean begging intensity of their offspring, which were raised by foster parents (Kölliker et al. 2000). Indeed, this sex‐specific co‐adaptation has been interpreted to be due to maternal effects and the fact that females were more responsive to offspring (vocal) begging than males (see Fig. 1 in Kölliker et al. 2000).

**Figure 1:**
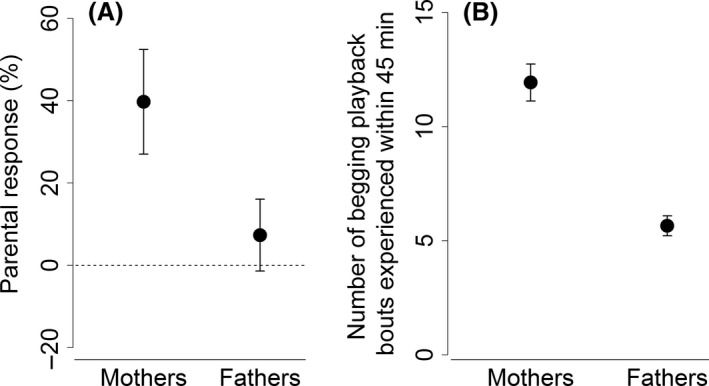
The parental response, defined as the proportional change in the provisioning rate from the silence treatment to the begging playback treatment (sensu Kölliker et al. 2000). A clear difference between the provisioning response of mothers and fathers (A). The dashed line indicates no change in parental provisioning between treatments, i.e. no response. Also the number of experienced begging playback bouts is different between the sexes (B). Given are mean ± SE.

Interestingly, males and females often play different roles during the phase of pre‐ but also postnatal parental care (Smiseth et al. 2012). Females in most nidicolous birds engage significantly more in nest sanitation (reviewed in Guigueno and Sealy 2012), thus, spending more time with their offspring. As a consequence, females may possess better information on offspring need, which may likely influence their responsiveness (Kölliker et al. 2000). These sex differences in nest attendance may significantly contribute to the previously observed sex difference in the parental response in provisioning as that manipulation allowed for different information access due to a constant frequency begging playback (Kölliker et al. 2000). Indeed, sensitivity to experimentally increased brood need was not different between the parents when both sexes had equal access to information – achieved via targeted begging playbacks, that is begging calls were played back to individual parents at each nest visit (Hinde 2006). However, these previous studies either neglected the consequences of differing sex roles (Kölliker et al. 2000) or, when allowing for equal information access, did not measure the consequences for parent‐offspring co‐adaptation (Hinde 2006). Thus, investigating whether it is information asymmetry between providing parents or a sex difference in responsiveness that leads to sex‐specific co‐adaptation is highly relevant.

We here investigated provisioning of blue tit (*Cyanistes caeruleus*) parents in response to begging playback and whether this response co‐varies (in a sex‐specific manner) with the begging behavior of their (cross‐fostered) offspring. As access to information may be unequal between the parents, we paid special attention to the individual perception of our manipulation and, further, we investigated how the consequences of differing sex roles during postnatal parental care impinge on parent‐offspring co‐adaptation. If sensitivity to vocal begging is equal for both sexes (sensu Hinde 2006), we expect to find parent‐offspring co‐adaptation independent of the parental sex.

## Material and Methods

### Study area and general methods

We conducted our experiments between March and May 2013 in a nest box population of blue tits breeding in Peerdsbos, a mature oak‐beech forest near Antwerp (51°16′27.73″N, 4°29′3.38″E, Belgium). We assessed clutch size and onset of incubation (to estimate hatch date of nestlings, see Table 1 for breeding parameters) via daily nest box checks (see also Lucass, Korsten et al. 2015a, 2015b). Studies on co‐adaptation require a disruption of potentially co‐adjusted offspring solicitation and parental provisioning behaviors, which was achieved by means of reciprocal cross‐fostering of whole clutches between two nests (=dyad), that were matched for hatch date (maximum difference two days) and clutch size (maximum difference two eggs) three days prior to hatching (see also Lucass, Korsten et al. 2015a, 2015b; Lucass, Stöwe et al.2015a, 2015b). Day of hatching was defined as day 1. On day 15, all nestlings were provided with a metal ring with a unique number and individually weighed (“fledgling mass”). Parents were caught on day 9 when feeding their foster nestlings using nest box traps. They were weighed, metal banded and provided with a unique color ring combination facilitating further identification. All experiments were conducted under licenses from the Ethical Committee for animals (ECD) of the University of Antwerp (license number 2011‐10).

**Table 1:** Breeding parameters for investigated nests (*N* = 40)

Average hatch date	16th May
Average brood size	10
Average begging score	7.28
Average provisioning rate (visits/min) of females (control treatment)	0.33
Average provisioning rate (visits/min) of males (control treatment)	0.36

### Begging behavior

On day 7, we took the second and fourth nestling in a descending weight rank and transferred them to a warmed artificial nest box to perform a begging test. Prior the test each nestling was fed with defrosted blue bottle maggots until satiation and begging behavior was video‐taped (Sony, DCR‐SX 30) after 60, 90, and 120 min of food deprivation by opening the nest box (Lucass, Korsten et al. 2015a, 2015b). Additionally, we played back a parental feeding call that was recorded in 2011 from an individual unrelated to all test nestlings. After testing, we immediately fed nestlings and returned them to their (foster) nest. From the videos we scored chick begging postures every second using an established rating scale (modified from Kilner 2002), ranging from 0 (chick is not begging) to 5 (chick's beak is open, the head is leaned back in a 90° angle and the back is in vertical position) and summed the scores afterwards. Brood begging intensity was calculated as the mean of all begging bouts (60, 90, and 120 min) of the two nestlings. Begging responsiveness was calculated as the change in average begging scores of the two nestlings across increasing levels of food deprivation by subtracting begging scores of 60 min from that of 120 min.

### Playback treatments and parental provisioning behavior

Before the start of our experiment we recorded begging calls of a brood (from a distant population: Wilrijk, Campus Drie Eiken, 51°09′49.729″N, 4°24′3.241″E, Belgium). The recordings were performed on day 11 post‐hatching, and the respective brood (10 nestlings) was food deprived for 1 h by blocking the nest box entrance. Begging calls were subsequently recorded with a sound‐recording unit (M‐Audio MicroTrack 24/96 Professional Mobile Digital Recorder).

Using the program Audacity (v. 2.0.0, Audacity Team) we created audiofiles at 32 bit and 44.1 kHz. The first 30 min were filled with silence. This was followed by a period of “begging call treatment” during which a begging call sequence (20 s) was played back every 90 s for 1 h. The “begging call treatment” was followed by a “silence treatment”, which represented 1 h of silence. We created a second audio‐file where the order of “begging call treatment” and “silence treatment” was reversed and audiofiles were randomly allocated to nests to stratify potential time of day effects.

On day 11, we placed a mini‐speaker with an inbuilt player (Difrnce SP 120) in 60 nest boxes that were equipped one day earlier with an infra‐red camera with an inbuilt microphone (420TVL) facing downwards to the nest to video‐tape parental provisioning behavior. The mini‐speaker was connected to a USB‐stick with one of the two audiofiles. We discarded the first 30 min of the videos in order to avoid feeding bias due to our disturbance (Kölliker et al. 1998). We also discarded the first 15 min of each treatment (begging call and silence) enabling parents to adjust their provisioning in response to the treatment. This resulted in videos of 45 min in each treatment that were analysed using “The Observer XT” (version 10.0.526, 2010; Noldus Information Technoloy, Wageningen, The Netherlands). We scored parental provisioning rates (visits/min) and the number of experienced begging playback bouts individually for the sexes. The individual parental response in provisioning was calculated as the difference between the provisioning rates during the begging call treatment and silence treatment. According to previous studies in closely related great tits (*Parus major*), nestling begging is unaffected by begging playbacks (Kölliker et al. 2000; Hinde 2006). Also, from the videos we observed that nestlings did not adjust their begging toward playback, but we did not quantify this in detail. In nestlings of tree swallows (*Tachycineta bicolor*) a slight increase in the number of begging offspring has been found, when the begging playback is targeted to the arrival of a parent (Leonard et al. 2009). Due to technical problems (camera/speaker, *N* = 12) or uniparental care (*N* = 8) we had to discard 20 nests, resulting in a sample size of 40 nests.

### Statistical analyses

At first we investigated whether pair members differed in their response to the begging playback. We performed a linear mixed effect model (LME) with the response in provisioning as dependent and parental sex as explaining variable. We included the random effect Nest ID nested in Dyad ID (model 1). In a second LME, using the same random effects as before, we investigated whether the number of begging playback bouts that birds experienced differed between the sexes (model 2). Finally, to fully disentangle factors driving the parental response we used an LME (random effect: see above) with the fixed effects parental sex, number of experienced begging playback bouts and the interaction of the two latter (model 3).

Next, we investigated patterns of co‐variation between the parental response in provisioning and begging behavior of genetic and foster offspring. To this end, we first set‐up an LME similar to Kölliker et al. (2000) on the parental response in provisioning. Nest ID nested in Dyad ID was used as random effect. Fixed effects were parental sex, brood size, hatch date (as Julian date), begging intensity of genetic and of foster nestlings. Further, we included two interactions between the parental sex and begging intensity of genetic, respectively foster nestlings (model 4), to check whether parents responded differentially.

As we found that the parental response in provisioning was not different between the sexes but rather dependent on the number of experienced begging playback bouts (see model 3), we added the number of experienced begging playback bouts as covariate to model 4 and further included an interaction term between sex and the number of experienced begging playback bouts (model 5).

In a last step, we repeated model 5 but replaced mean values with responsiveness of begging (model 6).

All statistical tests were performed in R, version 3.0.2 (R Core Team 2013). For implementing LMEs, we used the package “lme4” (Bates et al. 2013). To obtain a minimal model, we performed a stepwise backwards elimination by using the package “lmerTest” (Kuznetsova et al. 2014) that sequentially deletes terms with a *P*‐value higher than 0.05, starting with the least significant interaction. We confirmed the validity of all final models by visual inspection of residuals for normality, heteroscedasticity and non‐linear patterns.

## Results

### Determinants of individual parental provisioning response

Parents differentially adjusted their provisioning behavior in response to the playback treatment (LME1: *F*_1,39.0_ = 7.256, *P* = 0.010), with females responding more strongly than males (Fig. 1A). The sexes also differed in the number of begging playback bouts they were subjected to when being in the nest box (LME2: *F*_1,39.0_ = 46.399, *P* < 0.001), with females being significantly more exposed (Fig. 1B). Considering the latter revealed that the parental response in provisioning was significantly influenced by the frequency of exposure to the begging playback (LME3: *F*_1,52.31_ = 13.868, *P* < 0.001), but not by parental sex (*F*_1,47.53_ = 0.213, *P* = 0.647) or the interaction of the latter two (*F*_1,73.53_ = 0.175, *P* = 0.677).

### Co‐variation between parental response in provisioning and offspring begging behavior

When not taking the number of individually experienced begging playbacks into account, the begging intensity of genetic nestlings appeared to be marginally significant (model 4, *F*_1,38.0_ = 4.263; *P* = 0.046, Table 2), but this relationship did not differ between parents (model 4, *F*_1,37.0_ = 0.168; *P* = 0.685, Table 2). However, the parental response to playback differed between mothers and fathers (model 4, sex: *F*_1,39.0_ = 7.256; *P* = 0.010, Table 2).

**Table 2:** LME model (4) explaining the influence of offspring begging intensity on the parental provisioning response (without correcting for the effectiveness of the treatment). Nest ID nested in Dyad ID was included as a random effect. Significant variables that retained in the reduced model are highlighted in bold. *N* = 40 nests

	*F*	df	*P*
**Parental sex**	7.256	1,39.0	0.010
Brood size	0.930	1,35.65	0.312
Hatch date	0.963	1,37.0	0.333
**Begging intensity of genetic nestlings**	4.263	1,38.0	0.046
Begging intensity of foster nestlings	0.153	1,34.49	0.698
Parental sex × begging intensity of genetic nestlings	0.168	1,37.0	0.685
Parental sex × begging intensity of foster nestlings	0.272	1,38.0	0.605

**Figure 2:**
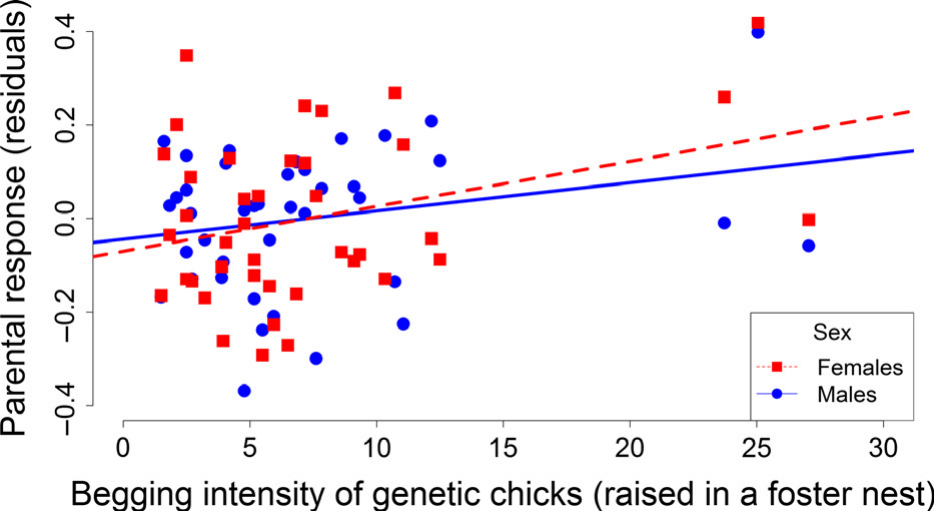
The relationship between begging intensity (measured as begging posture) of genetic nestlings (that were raised in a foster nest) and the residuals of the parental provisioning response (as the difference between provisioning rates toward the playback treatment and the control treatment) after controlling for the number of begging playback bouts that an individual parent experienced.

The significant effect of sex on provisioning response again vanished when the number of begging playbacks that individual birds experienced was included in the analysis (model 5, sex: *F*_1,48.11_ = 0.092; *P* = 0.761; number of experienced begging playback bouts: *F*_1,53.82_ = 15.678; *P* < 0.001; Table 3). Furthermore, the relationship between begging intensity of genetic nestlings and parental provisioning response became stronger (*F*_1,38.13_ = 5.595; *P* = 0.023, Table 3, Fig. 2).

**Table 3:** LME model (5) explaining the influence of offspring begging intensity on the parental provisioning response (correcting for the effectiveness of the treatment). Nest ID nested in Dyad ID was included as a random effect. Significant variables that retained in the reduced model are highlighted in bold. *N* = 40 nests

	*F*	df	*P*
Parental sex	0.092	1,48.11	0.761
Brood size	0.307	1,37.58	0.583
Hatch date	1.156	1,36.95	0.289
**Begging intensity of genetic nestlings**	5.595	1,38.13	0.023
Begging intensity of foster nestlings	0.532	1,36.16	0.471
Parental sex × begging intensity of genetic nestlings	0.564	1,36.64	0.458
Parental sex × begging intensity of foster nestlings	1.025	1,37.91	0.318
Parental sex × number of begging playback bouts experienced	0.476	1,70.79	0.492
**Number of begging playback bouts experienced**	15.678	1,53.82	<0.001

When considering chick begging responsiveness instead of begging intensity (i.e. mean values) the relationship was similar, but no longer statistically significant (model 6, begging responsiveness of genetic nestlings: *F*_1,38.40_ = 3.794; *P* = 0.059; Table 4).

**Table 4:** LME model (6) explaining the influence of offspring begging reaction norm on the parental provisioning response (correcting for the effectiveness of the treatment). Nest ID nested in Dyad ID was included as a random effect. Significant variables that retained in the reduced model are highlighted in bold. *N* = 40 nests

	*F*	df	*P*
Parental sex	0.160	1,47.58	0.691
Brood size	0.047	1,35.63	0.829
Hatch date	0.189	1,35.92	0.666
Begging responsiveness of genetic nestlings	3.794	1,38.40	0.059
Begging responsiveness of foster nestlings	0.862	1,37.53	0.359
Parental sex × begging responsiveness of genetic nestlings	1.316	1,36.52	0.259
Parental sex × begging responsiveness of foster nestlings	2.301	1,37.59	0.138
Parental sex × number of begging playback bouts experienced	0.0	1,68.57	0.993
**Number of begging playback bouts experienced**	14.454	1,52.65	<0.001

Brood size, hatch date, begging intensity (respectively begging responsiveness) of foster nestlings or interaction terms with parental sex did not significantly contribute to the models (Tables 2, 3, 4).

## Discussion

In this study, we manipulated the brood demand as perceived by the parents via playback of nestling begging. We show that blue tit parents do not differ in their sensitivity toward offspring begging. But different sex roles lead to a divergent access to information about (manipulated) offspring need, which in turn impinged on the rate of parental care. The consequences of our findings for among other sexual conflict resolution and parent‐offspring co‐adaptation will be discussed.

### Parental response to begging playback

Our first analysis revealed that mothers strongly increased their rate of provisioning in response to our begging playback treatment, mimicking increased demand. Fathers on the contrary responded only very little to our manipulation. These results differ from the outcomes of most previous studies that investigated parental provisioning in response to a begging playback (Ottosson et al. 1997; Burford et al. 1998; Clark and Lee 1998; Wright 1998; MacGregor and Cockburn 2002). However, our results are consistent with a study that we in fact replicated in terms of the experimental set‐up but with a different though phylogenetically closely related species (Kölliker et al. 2000). The result of the latter study was interpreted as such that females are more responsive to offspring (vocal) begging than males. In contrast, no sex difference in parental response to begging playback was found in a later study on the same species, when applying a slightly different methodological approach, that is targeted playback of begging calls (Hinde 2006; Hinde and Kilner 2007). We here show that the different methodologies are most likely responsible for this discrepancy in study outcomes. We played back a sequence of begging every 90 s, thus, at a constant frequency and independent of the presence of an adult (sensu Kölliker et al. 2000). When taking the effective exposure of an adult to our playback into account, we show that the observed sex difference in provisioning is unlikely to reflect a sex‐specific responsiveness toward vocal begging cues and thus offspring need. It is rather due to sex differences in the number of begging playbacks an individual experienced when feeding the brood. Females experienced the begging playback bout more often (and thus increased provisioning rate more steeply) than males because of different sex roles during postnatal parental care. Paternal nest time per visit was short (on average 15 s) as they just stayed in the nest until the prey item delivered was swallowed by a nestling. The likelihood of being in the nest during the playback was thus comparatively low. Mothers in contrast did not only provide food, but they also sanitized the nest as has been shown in previous studies (Christe et al. 1996b); the time they spent per visit in the nest was therefore much longer (on average 55 s). This obviously increased the likelihood of experiencing our begging playback. To clarify, females respond more strongly to the treatment since they are more exposed to it, while the parental response per se is not different between the sexes. Indeed, Hinde (2006) ensured that only the focal parent was exposed to the playback treatment when entering the nest box, which, as mentioned above, resulted in similar responses of male and female parents in terms of increased provisioning, which has been confirmed in a later study (Hinde and Kilner 2007).

Due to different sex roles during nest keeping females possess better information on manipulated offspring need. This may, also under natural conditions, lead to a sex asymmetry in information about offspring need, since hungry offspring continue to beg after the prey item has been delivered or during nest cleaning (pers. observation) although at a lower frequency as in the experimental set‐up. A potential information asymmetry between parents is likely to have implications for among other the negotiation about care in the context of sexual conflict over parental care (Trivers 1972). Successful negotiation will depend on how well a parent is informed about partner care as well as about offspring need. Here, females appear to be in an advantageous position as males possess less information about brood need (due to the aforementioned short nest time). In order to compensate for that, the sex with less information (here, males) may use partner effort as a source for information to adjust its feeding behavior, resulting in matched provisioning rates between parents (Johnstone and Hinde 2006). The latter has been shown in great tits, where parents take turns visiting the nest (Johnstone et al. 2014), although it is yet unclear whether this mechanism is indeed driven by males following their partner's behavior. Here, however the paternal and maternal provisioning responses are not matched, which is likely due to the relatively short duration (1 h) during which the begging calls were broadcasted.

Finally, our playback consisted of a begging call of only one brood. This design is considered to raise pseudo‐replication issues (Hurlbert 1984; Kroodsma et al. 2001), that is when observed effects can potentially be due to specific attributes of the (in this case) begging playback instead begging calls in general. Although this is a valid objection, we chose this design for two reasons: First, taking one begging call of a brood controls for variance between recordings. Second, pseudo‐replication plays an important role when it is important for an individual to recognize (attributes of) the sender of a “signal” (i.e. the playback), that is, for example, in interactions in a sexual context or during competition (see also example in McGregor 2000). However, begging vocalizations in blue tits function as a tool to communicate hunger and parents are unable to recognize a brood from its begging, which is supported by the fact that parents care for a brood even after cross‐fostering (see e.g. Lucass, Korsten et al. 2015a, 2015b). Thus, we believe that pseudo‐replication effects are negligible in our study (see also Burford et al. 1998).

### Co‐adapting parent‐offspring behaviors

Taking sex differences in access to manipulated information, thus the effectiveness of the treatment, here begging playback, into account, we found that the provisioning response of both parents co‐varied positively with begging intensity of genetic offspring that were raised in a foster nest. This relationship was much weaker when the effectiveness of the treatment was not included. The matching trait combinations of offspring begging intensity and parental provisioning response are likely to be the evolutionary outcome of selection that shaped family interactions to minimize fitness costs. However, previously observed sex‐specific mother‐offspring co‐variation in great tits (Kölliker et al. 2000) may rather represent an artifact of the methodology via a sex difference in access to playback. Thus, the hypothesis for biased mother‐offspring co‐adaptation that may be facilitated by maternal effects remains as yet unsupported (Hinde et al. 2009). We further show that such sex‐differences are not facilitated by the fact that mothers are more responsive to offspring need, which was in fact not the case in our study.

The results of this study are, however, in contrast with a previous study performed in the same population of blue tits showing negative co‐variation between paternal but not maternal provisioning responsiveness to changes in brood size and their offspring begging responsiveness to food deprivation (Lucass, Korsten et al. 2015a, 2015b). This may, again, relate to differences in the methodology. Here, a playback treatment of one begging cue was used, which was in addition a discontinuous manipulation (i.e. begging vocalizations are not played back at *every* parental visit). However, begging is a multicomponent signal (e.g. Leonard et al. 2003) and parents may use more than one begging cue (e.g. a visual cue like posture) to reliably assess nestling condition (“redundant signal” or “backup signal hypothesis” Johnstone 1996), as well as to respond to the number of offspring itself. Different begging signals may also carry different messages (i.e. not only hunger but also, for example, parasite infestation, see Christe et al. 1996a; “multiple message hypothesis”, Johnstone 1996). In contrast, brood size manipulations, as used by Lucass et al. (2015b), represent a constant manipulation (i.e. equally strong manipulation at every parental visit) of all begging cues, vocal and visual, where both parents respond to (e.g. Hegner and Wingfield 1987; Dijkstra et al. 1990; Verhulst and Tinbergen 1997; Sanz and Tinbergen 1999; Magrath et al. 2007). Thus, it appears vital for the interpretation to understand what information a given begging signal conveys, in order to understand the parental response, as well as the co‐variation with offspring traits.

Previous work suggested that the dynamic nature of the reciprocal interplay between parental provisioning and offspring begging should lead to co‐adapting behavioral reaction norms (Smiseth et al. 2008; Dobler and Kölliker 2009). We therefore additionally analysed the parental response to playback in terms of a change in feeding rate in relation to the begging behavior in response to an increase in hunger, both representing simple behavioral reaction norms. The co‐variation remained qualitatively similar (statistical trend for a relationship between both measures). However, our measure of begging intensity is in fact tightly linked (positive) to begging responsiveness (regression model: *F*_1,38_ = 25.65, *R*² = 0.40, *P* < 0.001). This is not surprising taking into consideration that begging intensity of nestlings after 60 min of food deprivation is low (i.e. a low begging score). Thus, it may be possible that the mean values (i.e. begging intensity) already captured the reaction norm of begging.

## Conclusions

We found that sex‐specific parental roles during the phase of parental care have the potential to affect the amount of information mothers, respectively, fathers can access about offspring need. This had clear effects on the outcome of our study. But it may also impinge on the resolution of evolutionary conflicts of interest within the family such as the sexual conflict over the amount of care to be provided. An information asymmetry about offspring need between pair members may likely affect negotiation.

However, parents did not differ in their responsiveness toward offspring need per se. Different responses toward manipulated offspring need (obtained via begging playbacks with a constant interval) are instead affected by different sex roles during parental care. Studying aspects of family life thus requires to consider (the importance of) different roles of all family members and to not only carefully fine‐tune the applied methodology toward the study aims but also for the interpretation of data. The latter also appears crucial for our understanding of co‐adaptation of offspring begging and parental provisioning.

## Conflict of Interest

The authors declare that they have no conflict of interest.
